# Primary Adenosquamous Carcinoma of the Prostate with Rectal Invasion

**DOI:** 10.1155/2022/7613482

**Published:** 2022-06-24

**Authors:** Pierre Azzi, Dominique Bossé, Ilias Cagiannos, Paul Borowy-Borowski, David Tiberi

**Affiliations:** ^1^Faculty of Medicine, University of Ottawa, Ottawa, Ontario, Canada; ^2^Division of Medical Oncology, The Ottawa Hospital Cancer Center, Ottawa, Ontario, Canada; ^3^Division of Urology, The Ottawa Hospital, Ottawa, Ontario, Canada; ^4^Department of Pathology and Laboratory Medicine, The Ottawa Hospital, Ottawa, Ontario, Canada; ^5^Division of Radiation Oncology, The Ottawa Hospital Cancer Center, Ottawa, Ontario, Canada

## Abstract

Prostate adenosquamous carcinoma (pASC) is a rare form of prostate cancer accounting for <1% of all cases. It is generally considered an aggressive variant often presenting with significant symptom burden and/or metastatic disease. Given its rarity, optimal management of this cancer is unknown. We present a case of a patient with pASC treated with radiotherapy and chemotherapy with excellent symptomatic improvement and local control.

## 1. Introduction

Prostate cancer is the most common malignancy (excluding skin cancers) in men accounting for 20% of all new cancer cases in men [1]. The vast majority of prostate cancers (roughly 95%) are of the adenocarcinoma subtype [2].

Prostate adenosquamous carcinoma (pASC) is a rare subtype accounting for <1% of all prostate cancers. It is a very aggressive malignancy that frequently metastasizes to the bone, liver, and lung [3].

Data regarding the optimal management of pASC is lacking given its rarity.

## 2. Case Presentation

We present the case of a 62-year-old man who presented to his family physician with lower urinary tract symptoms (LUTS), rectal bleeding, and pain. A digital rectal exam (DRE) was performed which revealed a bulky and indurated prostate, and the patient was referred to urology for investigation and placement of a urinary catheter.

Total prostate-specific antigen (PSA) was 3.11 ng/ml and urinalysis was negative. A staging workup consisting of computed tomography (CT), bone scan, and pelvic magnetic resonance imaging (MRI) was ordered.

**Figure 1:**
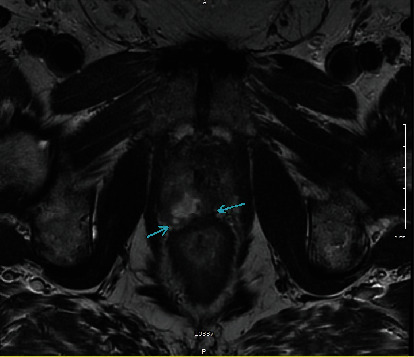
Axial T2-weighted MRI image showing the caudal aspect of the tumor abutting the caudal aspect of the rectum/upper aspect of the anal canal with possible serosal invasion.

Bone scan revealed no evidence of skeletal metastases. Pelvic MRI showed extensive partially necrotic right-sided prostatic carcinoma with definite extracapsular spread, seminal vesicle invasion, and involvement of the neurovascular bundles. There was also invasion into the mesorectal fat with suspected involvement of the caudal aspect of the rectal serosa/upper aspect of anal canal (Figure 1). CT of the chest, abdomen, and pelvis demonstrated a 2.8 cm mass centered in the right peripheral prostate, inseparable from the right seminal vesicle and abutting the anterior rectal wall. There was no evidence of distant metastatic disease.

**Figure 2:**
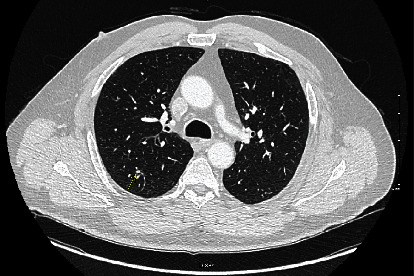
Representative histopathologic slides from transrectal prostate biopsy showing squamous cell carcinoma intimately mixed with glandular structures encircling a nerve (×400 magnification). IHC: immunohistochemistry; DPIN4 cocktail consisting of racemase, Ker903 (high-molecular weight cytokeratin), and p63.

The patient underwent transrectal ultrasound-guided prostate biopsy (TRUS) which revealed a poorly differentiated squamous cell carcinoma moderately that was positive for p63 and Ker903 (Figure 2). This was present in all 16 of the sampled cores and periprostatic fat invasion was present. There was a very small component of synchronous Gleason 6 acinar adenocarcinoma in 2 out of the 16 cores, representing at most 1% over the overall sampled tissue. The patient also had a cystoscopy to investigate for possible urethral mass or bladder mass which did not reveal any suspicious lesions.

This case was presented at our institutional tumor board for discussion. Pathology review by our dedicated genitourinary pathology team concluded that this was likely an adenosquamous carcinoma due to the small acinar adenocarcinoma component.

Due to the locally invasive primary tumor invading the rectum, the patient was deemed inoperable. The proposed management plan consisted of local radiotherapy followed by chemotherapy.

External beam radiotherapy using volumetric modulated arc therapy (VMAT) to a dose of 40.05 Gy in 15 fractions was administered to the pelvic disease. Within 10 days of starting radiotherapy, the rectal bleeding had stopped, and the patient began having a significant reduction in his rectal/pelvic pain.

**Figure 3:**
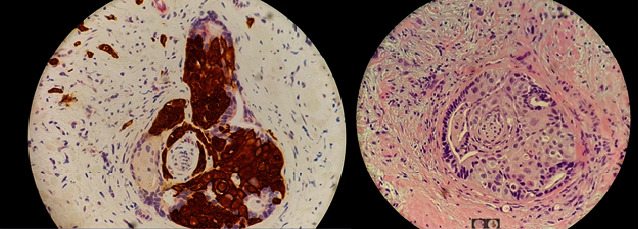
Axial chest CT scan showing a 5 mm solid pulmonary nodule suspicious for metastatic disease.

Restaging CT scans 2 months later demonstrated response at the level of the primary lesion treated with radiotherapy. Unfortunately, there was evidence of a new lung nodule suspicious for metastasis (Figure 3). The patient was started on docetaxel chemotherapy at a dose of 75 mg/m^2^ intravenously every 3 weeks. Follow-up CT scans 3 months after starting chemotherapy demonstrated disease progression with new, bilateral lung nodules and a new liver lesion consistent with metastatic disease. There was no evidence of bony disease on bone scan and PSA levels remained <4 ng/ml.

Currently, the patient is jointly followed by our institution's outpatient palliative care clinic in order to optimize management. Now, nearly 9 months from initial presentation his symptoms are much improved. His rectal pain has diminished significantly since radiotherapy and his narcotic medication has been tapered down and he no longer requires breakthrough doses. He was able to have his urinary catheter removed.

## 3. Discussion

Prostate adenosquamous cell carcinoma is a rare form of cancer that makes up 0.5%-1% of all prostate carcinomas. Only 25 cases were reported in the Surveillance, Epidemiology, and End Results (SEER) database [4]. Useful histopathological tests can include stains such as Ker903, whose presence rules out typical prostate adenocarcinoma and usually stains tumors with squamous differentiation [5]. These tumors can also have negative staining for PSA and PAP (prostatic acid phosphatase).

pASC patients often present with symptoms including dysuria, rectal/pelvic pain, acute urinary retention, urinary tract infection, hematuria, and bony pain due to metastases. These tumors can also have normal serum PSA and PAP. Risk factors for the development of prostatic adenosquamous carcinoma can include prior radiation and/or hormonal therapy [6], none of which our patient had received. There have been several documented cases of pASC in the absence of hormonal or radiotherapy, and some authors have suggested that this may reflect pluripotent cells capable of multidirectional differentiation [7].

Unfortunately, these forms of prostate cancer carry a poor prognosis with a median survival estimated at 12-14 months [6]. The disease is also frequently metastatic at diagnosis most commonly spreading to bones and lymph nodes.

Due to the small number of cases present in the literature, optimal treatment for this malignancy is controversial. Hennessey et al. recently reported a case of de novo pASC treated with a multimodal approach including surgery, androgen-deprivation therapy (ADT), docetaxel chemotherapy, and radiation with a good radiologic and clinical improvement 20 months post treatment [7].

Due to the lack of data regarding systemic therapy regimens, it is unclear what the expected response rate to chemotherapy or what the role of immunotherapy may be. Some have hypothesized that response to ADT is poor when there is a predominant squamous carcinoma component in the tumor. As a result, we elected not to prescribe ADT in our case due to the very minimal adenocarcinoma component.

Little et al. used an aggressive surgical approach, radical cystoprostatectomy, to treat 2 patients. One of the patients remained cancer free 40 months after initial diagnosis, and the other died 25 months after diagnosis from lung metastases [8].

Our patient was deemed inoperable due to the rectal involvement after tumor board review, so a decision to use sequential radiotherapy followed by chemotherapy was made. Due to the very significant bulk of local disease, the tumor board felt that radical chemoradiation would be unlikely to cure the patient and recommended a more moderate approach to balance local control and toxicity. Therefore, we selected a 3-week course of radiotherapy with a total dose (40.05 Gy) which is higher than what would typically be used for pure palliation but slightly lower than for radical-intent therapy. This treatment provided excellent symptomatic relief with an improvement in quality of life.

Based on the cases presented in the literature, radiation therapy and chemotherapy may be a useful multimodal approach for locally advanced and metastatic squamous cell carcinoma. Continued follow-up of our patient is needed to determine overall symptomatic benefit more long-term.

## 4. Conclusion

Prostate adenosquamous carcinoma is a rare and aggressive malignancy with a paucity of data to guide management. Published case reports suggest that surgery is reasonable for early-stage disease, while chemotherapy +/- radiotherapy may be used for more advanced stages. We present a case of pASC treated with sequential radiotherapy and multiagent chemotherapy with excellent symptomatic relief and local control.

Hopefully, continued follow-up of our current case and future case series will help refine the optimal management of pASC.

## Data Availability

No data were used.
